# Determination of Neuraminidase Kinetic Constants Using Whole Influenza Virus Preparations and Correction for Spectroscopic Interference by a Fluorogenic Substrate

**DOI:** 10.1371/journal.pone.0071401

**Published:** 2013-08-15

**Authors:** Bindumadhav M. Marathe, Vincent Lévêque, Klaus Klumpp, Robert G. Webster, Elena A. Govorkova

**Affiliations:** 1 Division of Virology, Department of Infectious Diseases, St. Jude Children’s Research Hospital, Memphis, Tennessee, United States of America; 2 Virology Discovery, Hoffmann-La Roche Inc., Nutley, New Jersey, United States of America; 3 Department of Pathology, University of Tennessee, Memphis, Tennessee, United States of America; Centre of Influenza Research, The University of Hong Kong, Hong Kong

## Abstract

The influenza neuraminidase (NA) enzyme cleaves terminal sialic acid residues from cellular receptors, a process required for the release of newly synthesized virions. A balance of NA activity with sialic acid binding affinity of hemagglutinin (HA) is important for optimal virus replication. NA sequence evolution through genetic shift and drift contributes to the continuous modulation of influenza virus fitness and pathogenicity. A simple and reliable method for the determination of kinetic parameters of NA activity could add significant value to global influenza surveillance and provide parameters for the projection of fitness and pathogenicity of emerging virus variants. The use of fluorogenic substrate 2′-(4-methylumbelliferyl)-α-D-N-acetylneuraminic acid (MUNANA) and cell- or egg-grown whole influenza virus preparations have been attractive components of NA enzyme activity investigations. We describe important criteria to be addressed when determining *K_m_* and *V_max_* kinetic parameters using this method: (1) determination of the dynamic range of MUNANA and 4-methylumbelliferone product (4-MU) fluorescence for the instrument used; (2) adjustment of reaction conditions to approximate initial rate conditions, i.e. ≤15% of substrate converted during the reaction, with signal-to-noise ratio ≥10; (3) correction for optical interference and inner filter effect caused by increasing concentrations of MUNANA substrate. The results indicate a significant interference of MUNANA with 4-MU fluorescence determination. The criteria proposed enable an improved rapid estimation of NA kinetic parameters and facilitate comparison of data between laboratories.

## Introduction

Influenza viruses are important human respiratory pathogens that can cause seasonal epidemics and occasional pandemics [1]. The viral surface glycoproteins hemagglutinin (HA) and neuraminidase (NA) play important roles in the influenza virus life cycle, and thus in the infection and spread of influenza. The influenza virus HA binds to sialic acid–containing glycoconjugates on the surface of target cells and mediates viral entry by membrane fusion after a pH-triggered conformational change in the endosomal compartment. NA is a sialic acid–binding protein that cleaves terminal sialic acid residues from glycoconjugates, facilitating virion progeny release from infected cells [2]. NA also plays a role at the stage of virus invasion of the ciliated epithelium of human airways by removing decoy receptors on mucins, cilia, and cellular glycocalix, strong binding to which can hinder virus access to functional receptors on the surface membrane of target cells [3], [4]. The NAs of influenza A and B viruses are structurally similar and exist as tetramers comprising 4 identical subunits. Each subunit is composed of a stalk region, containing the transmembrane domain, which anchors the protein in the virus membrane, and a box-like globular head. Each monomer contains an active site present as a deep pocket on the distal surface, although the NA enzyme functions only as a tetramer [5], [6].

The NA and HA glycoproteins recognize the same ligands on the surface of susceptible cells, but have opposite functions of facilitating virus entry and virus release from target cells. Therefore, HA and NA activities must be balanced during the entry and egress steps of virus replication [7], [8]. This requirement for balanced HA and NA components on the virion may have played a role in the emergence of naturally occurring NA inhibitor–resistant H1N1 viruses in the 2007 and 2008 seasons [9], and has also been proposed to be a critical factor for efficient respiratory-droplet transmission of influenza virus, including the ability of the swine-origin pandemic 2009 influenza virus to spread effectively in the human population [10]. The requirement for balanced NA activity also helps determine overall fitness and transmissibility of viruses carrying NA inhibitor–resistant mutations [11]. A rapid method to measure specific sialic acid–binding affinity and NA enzyme activity could therefore allow more systematic monitoring of virus variants and help to better understand the correlation among sequence, NA activity, fitness, and transmissibility of influenza viruses.

Global influenza surveillance studies commonly measure the susceptibility of virus isolates to inhibition by NA inhibitors oseltamivir and zanamivir. The influenza NA phenotypic assay uses small synthetic sialic acid conjugates as substrates that produce either a luminescent or a fluorescent signal upon cleavage by NA. The luminescent assay uses the 2-dioxetane derivative of neuraminic acid substrate [12], whereas the fluorescent assay employs the 2′-O-(4-methylumbelliferyl)-N-acetylneuraminic acid (MUNANA) substrate [13].

These sensitive and specific assay formats have also been used to determine influenza virus NA kinetic parameters [14]–[19]. Enzyme sources used in these studies included whole influenza virus preparations, detergent-solubilized whole influenza virus preparations, purified NA heads obtained from protease-treated influenza virus preparations, and extracts from cells transiently expressing the influenza NA protein. Results obtained from these different studies are broadly similar, but reflect a level of variability that may represent intrinsic differences between virus strains or enzyme preparations or that may arise from the variety of conditions used. In addition, in several cases in which enzymatic rate saturation curves are shown, suboptimal fit of data and reduced rates at higher substrate concentrations are apparent. The heterogeneity in current datasets complicates the interpretation of apparent changes in kinetic parameters between virus strains, which might determine differences in fitness and susceptibility to inhibition by antiviral agents.

The widely recognized importance of performing global surveillance of susceptibility of influenza viruses to NA inhibitors has led to the establishment and distribution of robust, validated, and standardized phenotypic assays [20]–[22]. The establishment of a validated and standardized method to determine influenza NA enzymatic properties could improve the value of surveillance and allow the monitoring of influenza NA enzyme properties across various laboratories. In this study, we analyzed the factors required to adapt the MUNANA-based assays into a transferrable assay to determine the NA enzymatic parameters *K*_m_ and *V*_max_ in influenza viruses. In addition to standardizing the procedures to approximate initial rate conditions under substrate excess, we also propose a simple empirical method to correct the nonlinear inner filter effect (IFE) and fluorescence interference contributed by the assay components, in particular the MUNANA substrate.

## Materials and Methods

### Viruses and Cells

Influenza A/Puerto Rico/8/1934 (H1N1) (A/PR/8/34) and A/California/04/2009 (H1N1pdm09) (A/CA/04/09) viruses were obtained from the St. Jude Children’s Research Hospital repository. Influenza B/Wisconsin/01/2010 (B/WIS/01/10, Yamagata Lineage) virus was provided by the US Centers for Disease Control and Prevention. Influenza viruses were grown in the allantoic cavity of 10-day-old embryonated chicken eggs (Marshall Durbin, Birmingham, AL) for 48 h at 35°C (influenza A viruses) or at 33C (influenza B virus). Madin-Darby canine kidney (MDCK) cells were obtained from the American Type Culture Collection (Manassas, VA) and maintained in minimal essential medium (MEM) with 5% fetal bovine serum and an antibiotics/antimycotics mixture (100 U of penicillin, 0.1 mg of streptomycin, and 0.25 µg of amphotericin B/mL). The virus-containing allantoic fluid was stored at –80°C until use.

### Infectivity of Influenza Viruses

The infectivity of influenza viruses was determined in MDCK cells by the plaque assay. Briefly, confluent MDCK cells were incubated for 1 h at 35°C (influenza A viruses) or 33°C (influenza B viruses) with 10-fold serial dilutions of virus in 1 mL infection medium (MEM with 0.3% BSA and 1 µg/mL L-[tosylamido-2-phenyl] ethylchloromethylketone [TPCK]–treated trypsin (Worthington Biochemical Corporation, Lakewood, NJ). Cells were then washed and overlaid with freshly prepared MEM containing 0.3% BSA, 0.9% Seaplaque agarose (Lonza, Rockland, ME) with 1 µg/mL TPCK-treated trypsin, and incubated at 35°C (influenza A viruses) or 33°C (influenza B viruses) for 3 days. Plaques were stained with 0.1% crystal violet solution containing 10% formaldehyde, and the plaque-forming units (PFU) per mL were determined.

### Measurement of NA Activity

The NA activity of influenza A and B viruses was measured by a fluorescence-based assay using the fluorogenic substrate MUNANA (Sigma-Aldrich, St Louis, MO), based on the method of Potier et al. [13]. Substrate cleavage by the NA enzyme releases the fluorescent product 4-methylumbelliferone (4-MU) (Sigma-Aldrich, St Louis, MO). Fluorescence was measured every 60 s for 60 min at 37°C in a Synergy 2 multimode microplate reader (BioTek Instruments, Winooski, VT), using excitation and emission wavelengths of 360 nm and 460 nm, respectively, and a sensitivity setting of 60%. Under these conditions, blank samples of enzyme buffer generated a background signal of approximately 10 relative fluorescence units (RFU) and a dynamic range to detect increasing concentrations of 4-MU fluorescence over 5 orders of magnitude. Two-fold virus dilutions were prepared in enzyme buffer [32.5 mM of 2-(N*-*morpholino) ethanesulfonic acid (MES), 4 mM of calcium chloride, pH 6.5] and added (100 µL/well) in duplicate to a flat-bottom 96-well opaque black plate (Corning, Tewksbury, MA). After preincubation for 20–30 min at 37°C, the MUNANA substrate (separately pre-incubated for 20–30 min at 37°C) was added to all wells (50 µL/well) to achieve a final concentration of 100 µM. Immediately after adding the MUNANA substrate, the plate was transferred to a 37°C prewarmed Synergy 2 multimode microplate reader. The fluorescence signal from the enzyme buffer and MUNANA substrate alone in the absence of enzyme was subtracted as background from the signals obtained in the other wells. A standard curve was generated for each experiment using 4-MU diluted in enzyme buffer at final concentrations of 0.05 µM to 26.67 µM. Background-corrected RFU was converted to 4-MU concentration (µM) and used to determine the percentage of substrate consumed during the reaction. Enzymatic reactions were performed under conditions of ≤15% MUNANA substrate conversion to product during the reaction time.

### NA Enzyme Kinetics Assay

NA enzymatic parameters were determined in reactions performed in flat-bottom 96-well opaque black plates (Corning, Tewksbury, MA), influenza virus preparations as described above, and various MUNANA substrate concentrations. All assay components were pre-warmed for 20–30 min at 37°C. The reaction plates were shaken for 30 s on a MTS 2/4 digital microtiter shaker (IKA Works, Inc, Wilmington, NC) and transferred to a Synergy 2 multimode microplate reader (BioTek Instruments, Winooski, VT) prewarmed at 37°C. Fluorescence was monitored every 60 s for 60 min at 37°C, using excitation and emission wavelengths of 360 nm and 460 nm, respectively. Enzymatic reactions were performed under conditions where signal-to-noise ratios were above 10 during more than 30 min of the reaction time, and where corrected 4-MU fluorescence increased linearly for at least 30 min until the last time point. The inclusion of data with lower signal-to-noise ratios will result in increased variability and error. Time course data from each concentration of the MUNANA substrate were examined for linearity by linear regression analysis. Data with *R* ^2^>0.99 were used for analysis.

### Correction for Spectroscopic Interference in the MUNANA-based Fluorescence Assay

The spectroscopic overlap of absorption and emission between the MUNANA substrate and 4-MU product in the NA enzyme assay results in a MUNANA concentration–dependent nonlinear interference with 4-MU concentration determination in the assay. To adjust the relative fluorescence determined in the assay for this effect, a correction factor was empirically determined, using control measurements of fluorescence with 4-MU alone and in mixtures with MUNANA. The correction factor was calculated by dividing the fluorescence signal (in RFU) produced by 4-MU in the presence of different concentrations of MUNANA by the fluorescence signal produced by 4-MU alone (in RFU) (Table 1). The correction factors for each MUNANA concentration were then used to correct RFU values obtained from the NA enzymatic reactions performed at the equivalent MUNANA concentrations. The kinetic parameters *V*_max_ and *K*_m_ were calculated by fitting the corrected data to equation (1),
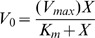
(1)where *V*_0_ is the steady-state rate of MUNANA substrate turnover by NA, *V*_max_ is the maximum rate (µM/min), *X* is the concentration of MUNANA (µM) in the reaction mixture and K_m_ is the Michaels constant (µM) at which the reaction rate is half of *V*_max_. Equation 1 was fitted to the data using the GraphPad Prism 5.0 software (GraphPad Software, La Jolla, CA).

**Table 1:** Calculation of the spectroscopic correction factor.

	Self-fluorescence (RFU)		Fluorescence of MUNANA plus 4-MU (RFU)		
Concentration of MUNANA (µM)	MUNANA^a^	4-MU^b^	Non-blanked^c^	Blanked^d^	Correction Factor^e^
Column 1	Column 2	Column 3	Column 4	Column 5	Column 6
2000	14340	10184	20871	6531	0.64
1000	8508	10430	16034	7527	0.74
500	4775	10244	13219	8445	0.83
250	2646	10264	11448	8802	0.86
125	1383	10176	10336	8953	0.88
62.5	742	10268	10015	9273	0.91
31.25	413	10126	10032	9619	0.94
15.63	242	10269	10142	9900	0.97
7.81	149	10182	9981	9833	0.96
3.91	101	10097	10078	9977	0.98
1.95	73	10201	10315	10242	1

**Abbreviations:** MUNANA, 2′-(4-methylumbelliferyl)-α-D-N-acetylneuraminic acid; 4-MU, 4-methylumbelliferone; RFU, relative fluorescence units.

a Fluorescence of MUNANA alone in enzyme buffer at 1.95–2000 µM concentrations (shown in column 1).

b Fluorescence of 4-MU alone in enzyme buffer at 19 µM concentration. Mean (± SD) 4-MU fluorescence in enzyme buffer was 10222±90 RFU.

c Fluorescence of 4-MU in enzyme buffer at 19 µM concentration in the presence of 1.95–2000 µM MUNANA concentrations (shown in column 1).

d Values (in RFU) of MUNANA fluorescence (column 2) subtracted from the values (in RFU) obtained in the mixture of 4-MU and MUNANA (column 4).

e Correction factor calculated as the ratio of 4-MU fluorescence (in RFU) in the mixture with MUNANA substrate (column 5) and 4-MU fluorescence (in RFU) alone (mean value from column 3).

### Statistical Analysis

The kinetic parameters *K*_m_ and *V*_max_ were compared by unpaired two-tailed *t*-test. A probability (P) value less or equal to 0.05 was prospectively chosen to indicate that the result was not attributable to chance.

## Results

### Selection of Influenza Virus Dilution for the NA Enzyme Kinetics Assay

We investigated options to adapt the MUNANA substrate–based NA phenotypic assay into a simple, fast, flexible, and transferrable influenza NA enzyme assay to determine kinetic constants. The assay format should allow standardization and comparison of data among different influenza virus isolates and among laboratories. We chose whole influenza virus preparations as the source of enzyme, as these are readily available in influenza surveillance laboratories. However, other sources of enzyme can likely be used for the procedure with minor modifications. The number of virions in influenza virus preparations and the amount of NA glycoprotein present on each virion can differ among influenza virus strains. Therefore, we used 3 measurable criteria to select the enzyme amount or virus dilution in order to provide an adequate fluorescence signal and to approximate steady-state turnover conditions: (1) RFU generated in the enzymatic reaction to be within the dynamic range of the plate reader and of the 4-MU product standard curve, with linear regression analysis of standard curves returning *R* ^2^ values greater than 0.99; (2) the amount of MUNANA substrate converted to product during the reaction to be 15% or lower; and (3) the signal-to-noise ratio of RFU during the reaction to be greater than 10. The corrected fluorescence readings (expressed in RFU) were converted into 4-MU concentrations (expressed in µM) based on the standard curve for each experiment. The detailed conversion of RFU values to 4-MU concentrations (µM) is shown in Table S1. The rate of an enzymatic reaction is dependent on the enzyme and substrate concentrations. To enable the use of a hyperbolic equation for the determination of kinetic constants (K*_m_* and V*_max_*), the enzymatic reaction needs to be adjusted to approximate initial rate conditions, i.e. conditions where the concentration of substrate remains approximately constant during the reaction time. In this case the impact of changes of the substrate concentration on the forward reaction rate and the reverse reaction are negligible. Typically, initial rate conditions can be approximated by maintaining substrate concentrations within 85–100% of the starting concentration. The smaller the amount of substrate used during the reaction, the closer initial rates can be approximated. The influence of changing substrate concentrations on the apparent reaction rate will become significant once substrate concentrations are changed by more than 15%.

**Figure 1:**
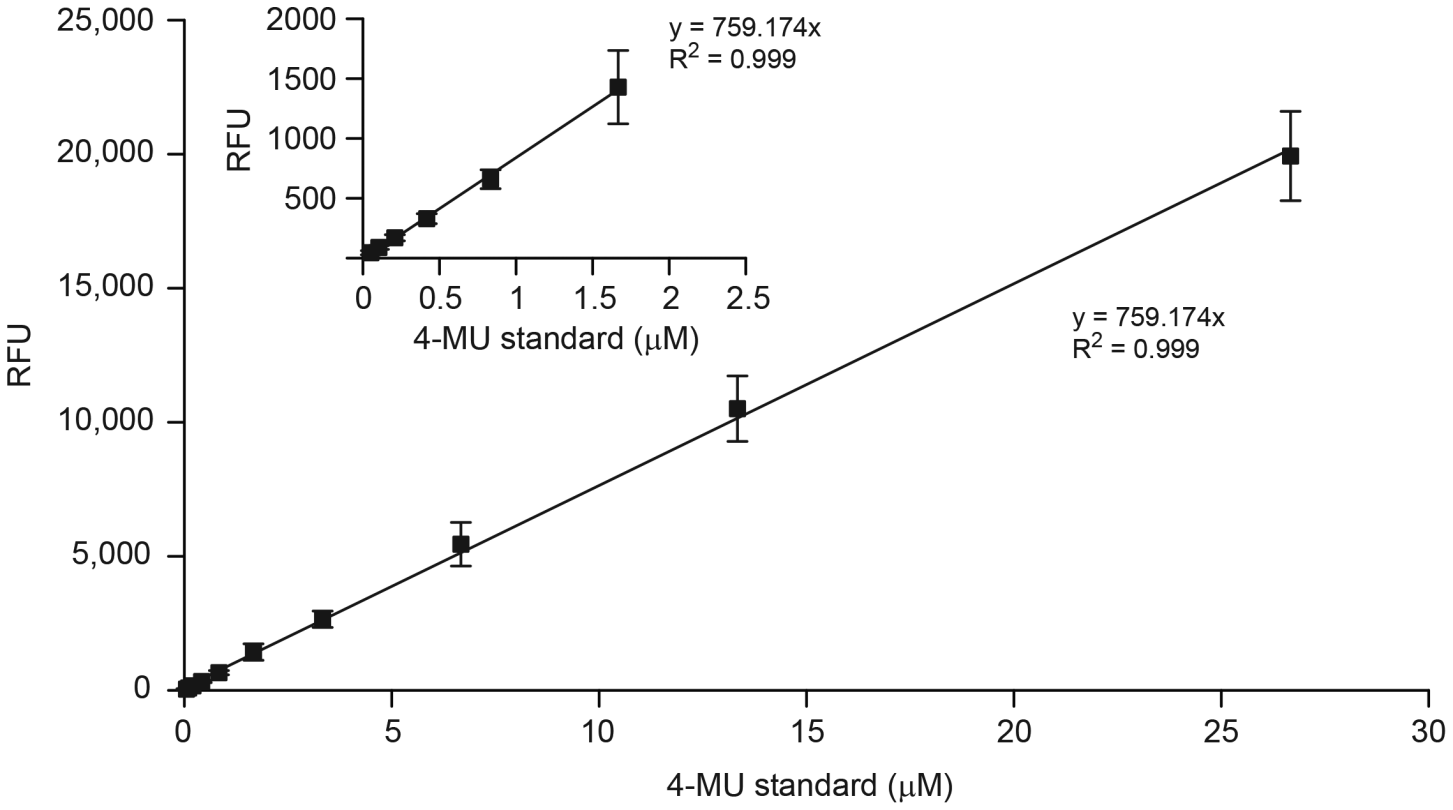
Standard curve of 4-methylumbelliferone (4-MU) fluorescence. Fluorescence intensity was measured using a Synergy 2 multimode microplate reader at excitation and emission wavelengths of 360 nm and 460 nm, respectively. Relative fluorescence units (RFU) obtained at low 4-MU concentrations (0–2.0 µM) are shown in the insert. Each data point represents the mean ± standard deviation (SD) of 10 independent measurements.

Figure 1 shows a standard curve of concentration-dependent 4-MU fluorescence. The final concentration of 4-MU (µM) in the enzyme buffer was plotted against RFU and analyzed by linear regression (*R* ^2^>0.99). There was a linear relationship between 4-MU concentration and fluorescence intensity, and there was no evidence of an inner filter effect (IFE) by 4-MU in the concentration range tested. The 4-MU standard curves were generated for all enzyme activity measurements in which the MUNANA substrate was used.

**Figure 2:**
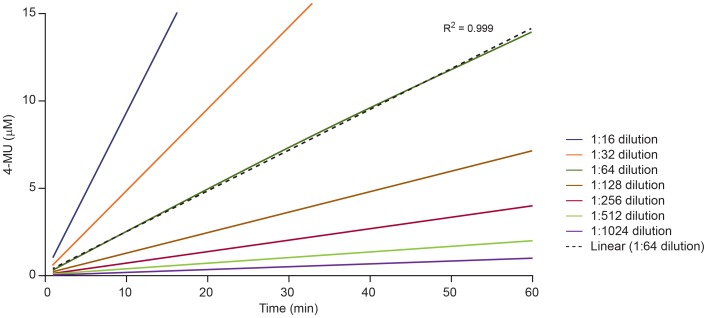
Influenza A/CA/04/09 (H1N1pdm09) virus dilution selection for determination of NA enzyme kinetics parameters. Two-fold dilutions of A/CA/04/09 (H1N1pdm09) virus were prepared in enzyme buffer. The graph shows virus dilutions (1∶16 to 1∶1024) that generated linearly increasing amounts of 4-MU over the reaction time (*R* ^2^>0.99). Fluorescence intensity was recorded every 60 s for 60 min at 37°C with the MUNANA substrate at a final concentration of 100 µM. Blank signal value determined from reactions without virus was subtracted from RFU generated in the NA enzymatic reactions. The virus dilution of 1∶64 was selected in this case for further studies on the basis of linearity of the curve (dotted line), signal-to-noise ratio ≥10, and conversion of ≤15% of the total amount of MUNANA after 60 min.

**Figure 3:**
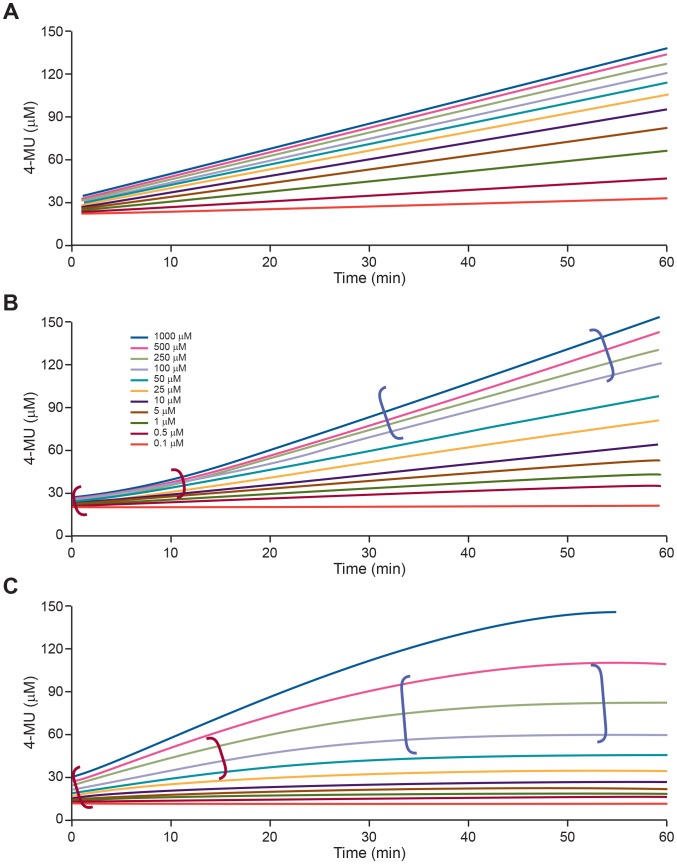
Possible kinetic profiles of NA enzyme reactions at different MUNANA substrate concentrations. Fluorescence intensity was recorded every 60 s for 60 min at 37°C with the MUNANA substrate at the final concentration of 0.1–1000 µM. (A) Profile consistent with steady-state conditions during the time of the reaction. (B) Dataset with initial lag phase (shown in red parentheses) and (C) datasets with lag (shown in red parentheses) and saturated phases (shown in blue parentheses) indicate suboptimal assay conditions and a requirement for assay optimization.

A virus dilution scan was performed for each new virus preparation, using a 2-fold dilution scheme up to 1∶1024 to identify virus dilutions that generated a proportional increase in concentration of 4-MU following incubation with MUNANA substrate (Figure 2). Different kinetic reaction progress curves may be obtained when NA enzymatic reactions are performed with different virus preparations and different MUNANA substrate concentrations. Figure 3 illustrates the possible kinetic profiles under different MUNANA substrate concentrations. Figure 3A shows the profile of a dataset that approximates steady-state conditions over the reaction time period at different MUNANA substrate concentrations. In contrast, profiles indicating a significant lag phase of the reaction (Figure 3B) or a combination of a lag phase and saturation (Figure 3C) may be obtained under suboptimal reaction conditions, and may reflect a lack of pre-incubation of the reaction components and equilibration to the reaction temperature or the use of an inappropriate virus dilution in the assay. Such profiles require adjustment of assay conditions before datasets are generated to determine NA kinetic parameters. With an appropriate virus dilution and under the assay conditions described, profiles similar to those shown in Figure 3A were consistently obtained with different influenza virus preparations and substrate concentrations spanning a range from 0.1-fold to 10-fold of the *K*_m_ value. A reference virus preparation of influenza A/PR/8/34 (H1N1) was also included in all experiments as a quality control. Enzyme datasets were accepted if the *K*_m_ and *V*_max_ values for the reference influenza A/PR/8/34 preparation did not vary from established values of the control batch by more than 10%.

### Data correction for MUNANA-associated Fluorescence and Inner Filter Effects

**Figure 4:**
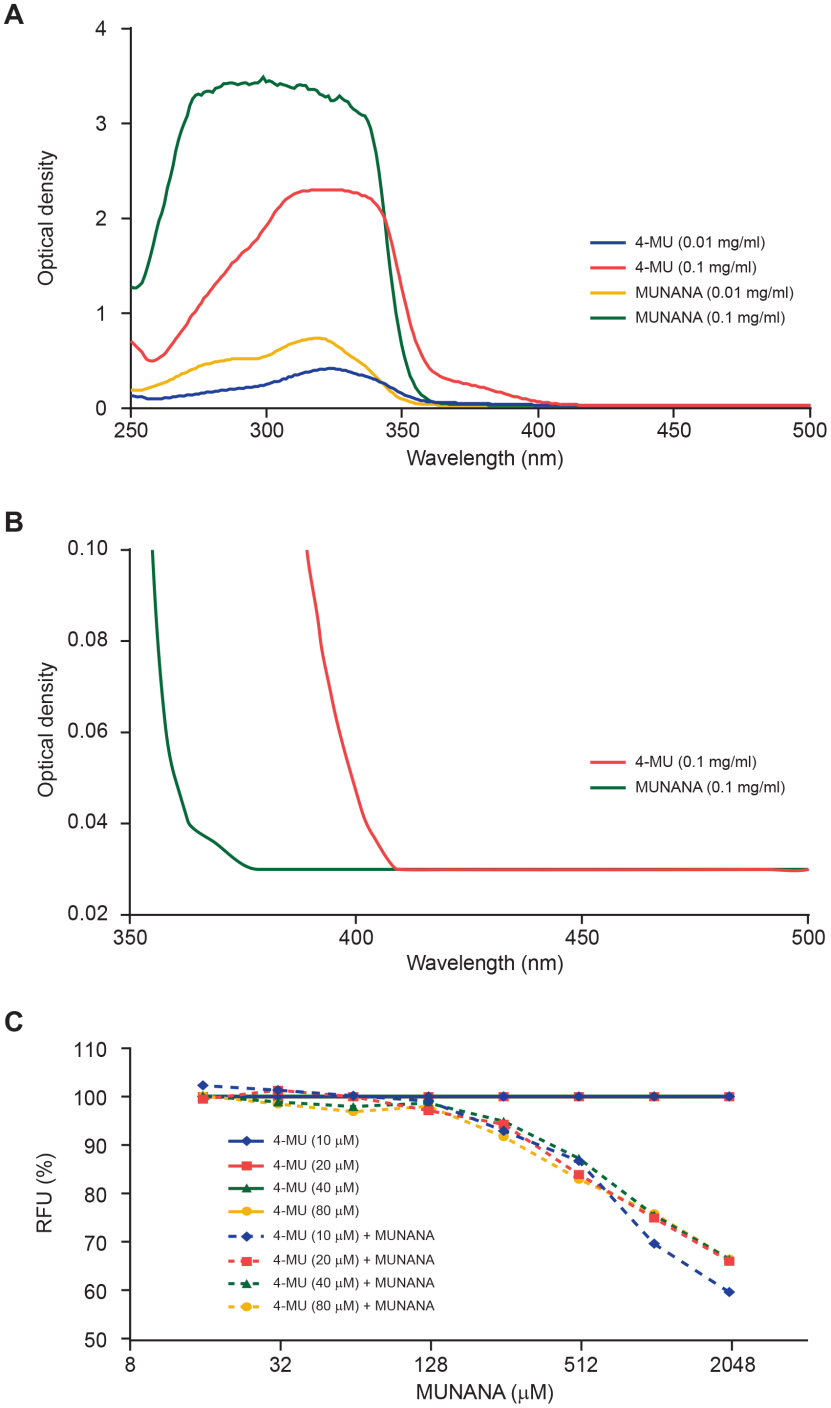
Inner filter effect (IFE) from light absorption at the excitation and emission wavelengths of the 4-MU product. (A) Absorbance spectra of MUNANA and 4-MU at 0.01 mg/mL and 0.1 mg/mL concentrations in enzyme buffer. Optical density was measured using Synergy 2 multi-mode microplate reader in a UV-transparent 96-well plate. (B) Absorbance spectra of MUNANA and 4-MU at 0.1 mg/mL concentration. (C) 4-MU fluorescence measured in the presence of different concentrations of MUNANA (15–2000 µM) shows similar impact of MUNANA-associated spectroscopic interference across 4-MU concentrations of 10–80 µM.

Fluorescence measurements can be affected by factors such as chemical composition of the solution, temperature, pH, and optical path lengths. Most of these factors can be approximated to be constant between control and test samples by controlling reaction conditions. However, molecules in the reaction that absorb light at the excitation or emission wavelength of the fluorophor that needs to be quantified can significantly affect fluorescence measurements. As shown in Figure 4, MUNANA and 4-MU had overlapping light absorption spectra at wavelengths between 250 nm and 500 nm. Light absorption of MUNANA at the 4-MU excitation and emission wavelengths was substantial when the fluorescence intensity of 4-MU was measured in the presence of increasing concentrations of MUNANA. Fluorescence emission from 4-MU solutions decreased significantly in the presence of MUNANA (Figure 4C). In addition, the MUNANA substrate itself showed significant fluorescence at the 4-MU excitation and emission wavelengths and at the MUNANA concentration range used in the NA enzyme activity assay, which caused substantial nonlinear interference with MUNANA and 4-MU fluorescence measurements (Table 1).

**Figure 5:**
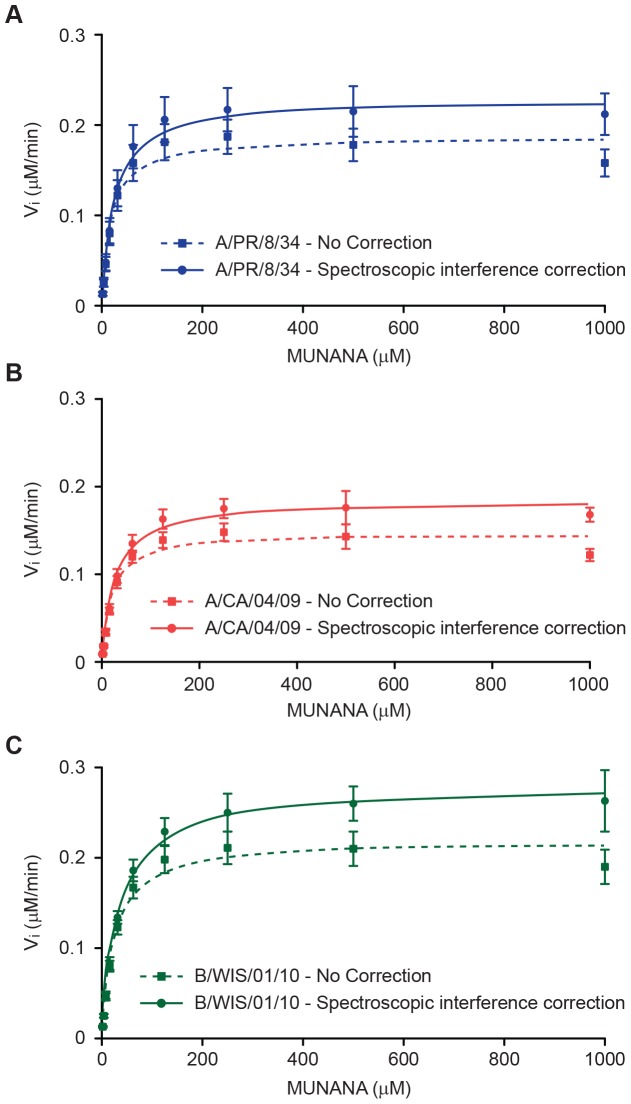
NA enzyme kinetics curves with and without spectroscopic corrections. (A) Data for A/PR/8/34 (H1N1) influenza virus; (B) A/CA/04/09 (H1N1pdm09) influenza virus and (C) B/WIS/01/10 influenza virus. Each data point represents the mean of 4 (A/CA/04/09 and B/WIS/01/10 viruses) or 7 (A/PR/8/34 virus) independent measurements. Dotted lines represent data without spectroscopic correction and solid lines represent data fitting to a hyperbolic equation after correction.

These spectroscopic interferences significantly affected data analysis in the MUNANA-based enzyme assay, especially when enzyme activity was determined across multiple MUNANA concentrations. The interference was apparent in experiments performed to calculate NA reaction velocity in the presence of increasing MUNANA concentrations as a reduction in enzyme activity at increasing substrate concentrations (Figure 5, dotted lines). This phenomenon was observed previously but was not identified as spectroscopic interference [23]–[26]. The impact of spectroscopic interference on data analysis can be minimized by determining correction factors from the addition of internal controls on the assay plate, similar to the method described by Liu and colleagues [27]. In our method, nonlinear interference of MUNANA at different concentrations is measured by comparing 4-MU fluorescence alone and in mixtures with MUNANA (Table 1).

Figure 5 shows results of adjusting RFU measurements in the MUNANA-based NA assay. Initial reaction velocities of the NA reaction were determined without (dotted lines) or with (solid lines) spectroscopic interference corrections for MUNANA interference. As seen in Table 1 and Figure 5, the nonlinear spectroscopic interference of MUNANA was detectable even at low micromolar concentrations of MUNANA, emphasizing the importance of data correction in this assay.

### Characterization of NA Enzyme Kinetics of Influenza Viruses

The kinetic parameters *K*_m_ and *V*_max_ were determined for 3 human influenza viruses by non-linear fitting of the fluorescence data after correction for spectroscopic interference. The corrections significantly improved the quality of data fitting (Figure 5), and there was a significant increase in *K*_m_ and *V*_max_ values after corrections were applied (*P*<0.05, unpaired two-tailed *t-*test) (Table 2).

**Table 2:** Neuraminidase enzyme kinetics parameters of influenza viruses.

		NA enzyme kinetics parameter			
		*V*_max_ (µM/min)		*K*_m_ (µM)	
Influenza virus	Infectivity^a^	Spectroscopicinterference corrected^b^	Increase fromuncorrected (%)^c^	Spectroscopicinterference corrected^b^	Increase fromuncorrected (%)^c^
A/PR/8/34 (H1N1)	8.7±0.05	0.23±0.03	23	25±3.3	34
A/CA/04/09 (H1N1pdm09)	7.3±0.09	0.19±0.01	26	28±3.4	34
B/WIS/01/10	7.6±0.02	0.28±0.03	28	34±3.1	41

a Virus infectivity was determined by plaque assay in MDCK cells. Values are expressed in log_10_ PFU/mL (mean ± SD) from 3 independent determinations.

b Results represent the means ± SD from 7 independent determinations for A/PR/8/34 (H1N1) virus and 4 independent determinations for A/CA/04/09 (H1N1pdm09) and B/WIS/01/10 viruses.

c Percent increase of values as calculated after the correction for spectroscopic MUNANA interference. The increases in *V*_max_ and *K*_m_ values after correction were statistically significant (*P*<0.05, unpaired two-tailed *t-*test).

The mean *K*_m_ values for 3 influenza viruses tested ranged from 25 µM to 34 µM, and the *K*_m_ values determined without the correction factors showed wider ranges between virus isolates than those determined using the correction factors. The mean *V*_max_ values for 3 influenza viruses ranged from 0.19 µM/min to 0.28 µM/min. The use of the correction factor resulted in a robust and repeatable enzyme assay for different virus preparations (Table 2).

## Discussion

**Figure 6:**
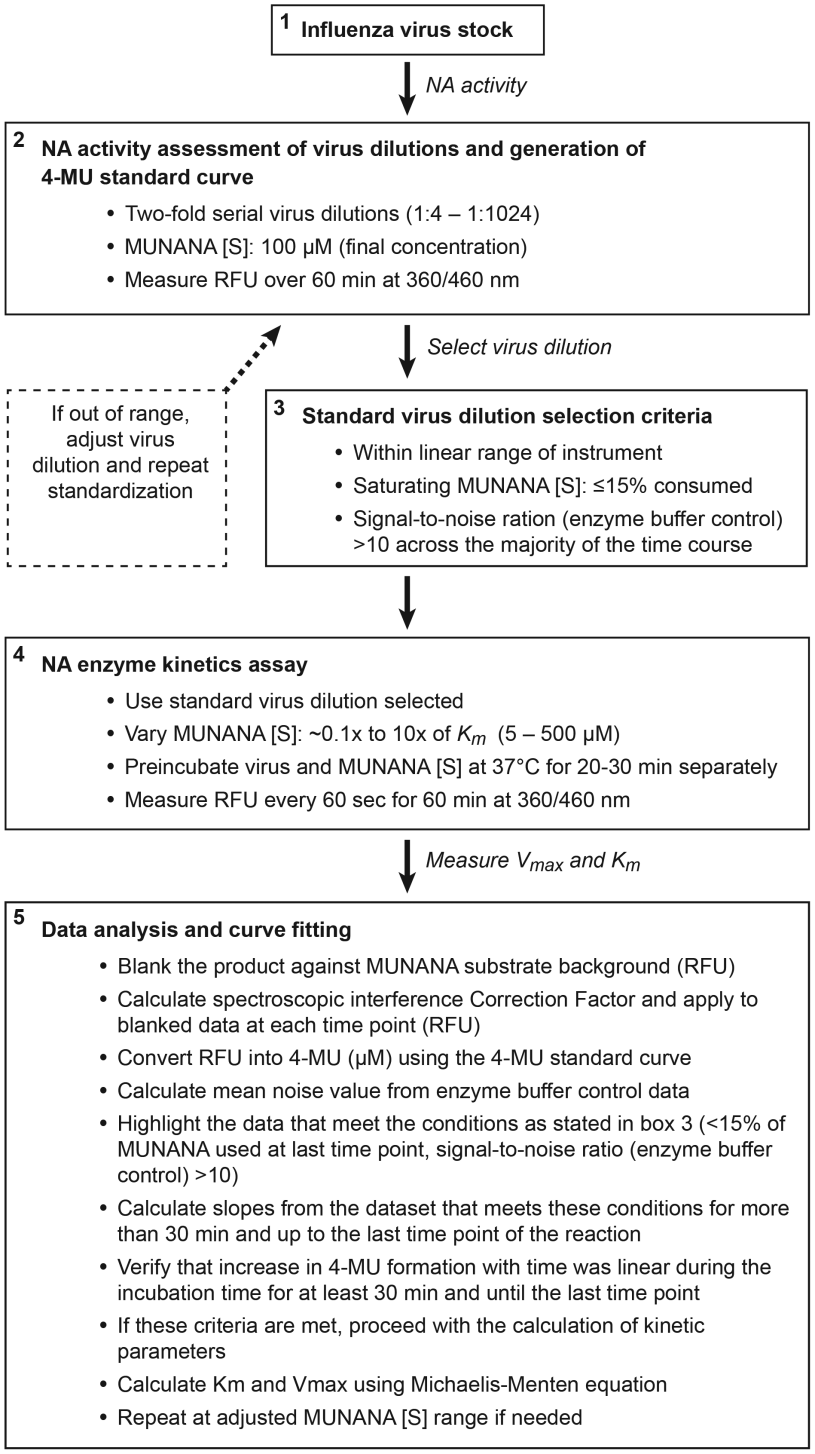
Flowchart for the determination of NA enzyme kinetic parameters using whole influenza virus preparations.

The NA protein plays an important role in the life cycle of influenza A and B viruses and is the target for a class of anti-influenza drugs, the NA inhibitors. The enzyme activity of NA and its interplay with HA is important to the overall fitness, transmissibility, and pathogenicity of influenza viruses [8], [11], [28], [29] and is also a determinant of barrier to resistance to NA inhibitors and the emergence of naturally drug-resistant virus variants. A more comprehensive monitoring of the evolution of NA enzyme activity in the context of newly emerging influenza viruses can improve influenza surveillance by eventually increasing our ability to predict the risk for emergence of transmissible, naturally occurring drug-resistant or more pathogenic influenza viruses [9], [16], [19], [30], [31]. Although the importance of NA enzyme efficiency and affinity has been previously discussed, the lack of a standardized method to measure NA enzyme kinetics has resulted in considerable variations in values of kinetic parameters determined at different laboratories. The MUNANA-based assay has been established as a phenotypic assay for the surveillance of influenza virus evolution and emergence of virus variants with altered susceptibility to inhibition by NA inhibitors by using a single MUNANA concentration [20], [32], [33]. The NA assay method described herein enables rapid estimation of enzymatic parameters across influenza virus isolates. This method is amenable to virology laboratories, addresses the important aspect of adapting a fluorescence-based method into a plate-based format, and can improve the comparison of data among laboratories. The method is summarized in Figure 6. In this study, we present the criteria used to adapt this assay format to estimate the kinetic constants of the NA enzyme of influenza viruses, which could lead to a more standardized approach of measuring enzyme activity in virology laboratories. The determination of kinetic parameters of NA activity requires the use of multiple concentrations of MUNANA and is therefore subject to nonlinear interference of 4-MU quantification by the spectroscopic interference associated with micromolar concentrations of MUNANA. Notably, a single concentration of MUNANA is used in the phenotypic assay for determination of susceptibility of influenza viruses to NA inhibitors, and the spectroscopic interference was similar across different 4-MU fluorescence tested. We therefore expect that antiviral IC_50_ values determined in the MUNANA based phenotypic assay will be similar with or without correction for the MUNANA spectroscopic interference with 4-MU fluorescence. We propose an empiric method to correct the spectroscopic interference contributed by the MUNANA substrate. This method determines the MUNANA interference with 4-MU quantification, based on the fluorescence analysis of mixtures of MUNANA and 4-MU that reflect the concentrations of MUNANA used in the enzyme assay. This real-time determination of RFU correction factors is easy to adapt to a microtiter plate enzyme assay format. The application significantly improved the data quality and repeatability of NA kinetic parameters measurements in our study.

The use of whole influenza virus preparations as a source of NA enzyme may be convenient for routine enzyme activity monitoring, as many virology laboratories are already using such preparations in NA inhibitor phenotypic assays. Also, selecting an appropriate virus dilution is critical to provide enzyme activity measurements that meet the requirements of steady-state substrate conversion during the reaction period with 15% or less of the substrate consumed. By our proposed method, we have determined *K*_m_ and *V*_max_ values for NA from 3 influenza A and B viruses with high reproducibility. It will be useful in the future to add to this protocol a method to determine NA protein concentration, which would allow assessment of enzyme efficiency for comparison among virus strains. A reliable method for NA quantification may require the use of a robust anti-NA antibody with limited interference from NA sequence polymorphisms and NA tetramer instability [34]–[36]. A mass spectrometric method has also been recently reported for simultaneous quantification of NA and HA protein in influenza virus preparations [37]–[39]. Such a procedure could be a very useful complement to NA enzyme activity monitoring.

Interestingly, although the kinetic parameters were similar for the 3 viruses used in this study, their infectivities in MDCK cells differed by up to 1.4 log_10_ PFU/mL. The pandemic influenza virus A/CA/04/09 (H1N1) showed the lowest infectivity in this system. It would therefore be interesting to investigate whether the NA enzyme activity was not in optimal balance with the HA binding affinity to sialic acid–containing substrates of the A/CA/04/09 (H1N1pdm09) virus, and whether therefore a mutation that could slightly reduce the NA enzyme activity could improve infectivity of this strain. It will also be useful to monitor the NA sequence evolution of H1N1pdm09 viruses with respect to harmonization of NA enzyme activity and infectivity in human target cells.

Comparative NA enzyme kinetics assays from whole virus preparation have been performed using equivalent doses of infectious virus particles [10], [23], [25]. In contrast, we propose here a selection of virus concentration that is solely based on its enzymatic properties to meet the requirements for steady-state kinetic analysis. Such data enable the study of the correlation between NA enzyme activity and infectivity as well as the contribution of other factors to infectivity across cell types.

In summary, we describe an improved method for the rapid determination of NA enzyme kinetics parameters of influenza viruses by using whole virus preparations on multiwell microtiter plates and correction for spectroscopic interference of the MUNANA substrate. The application of this method in the surveillance of influenza virus evolution, such as the adaptation of swine-origin H1N1 virus to humans or the evolution of NA in H5N1 viruses, could provide important information about the role of NA in influenza virus fitness, pathogenicity, and transmissibility.

## Supporting Information

Table S1 (XLS)
